# Multivariate BWAS can be replicable with moderate sample sizes

**DOI:** 10.1038/s41586-023-05745-x

**Published:** 2023-03-08

**Authors:** Tamas Spisak, Ulrike Bingel, Tor D. Wager

**Affiliations:** 1 Institute of Diagnostic and Interventional Radiology and Neuroradiology, University Medicine Essen, Essen, Germany; 2 Center for Translational Neuro- and Behavioral Sciences, Department of Neurology, University Medicine Essen, Essen, Germany; 3 Department of Psychological and Brain Sciences, Dartmouth College, Hanover, NH USA

**Keywords:** Neuroscience, Cognitive neuroscience, Learning algorithms

**arising from**: S. Marek et al. Reproducible brain-wide association studies require thousands of individuals. *Nature* 10.1038/s41586-022-04492-9 (2022)

Brain-wide association studies (BWAS)—which correlate individual differences in phenotypic traits with measures of brain structure and function—have become a dominant method for linking mind and brain over the past 30 years. Univariate BWAS typically test tens to hundreds of thousands of brain voxels individually, whereas multivariate BWAS integrate signals across brain regions into a predictive model. Numerous problems have been raised with univariate BWAS, including a lack of power and reliability and an inability to account for pattern-level information embedded in distributed neural circuits^1–4^. Multivariate predictive models address many of these concerns, and offer substantial promise for delivering brain-based measures of behavioural and clinical states and traits^2,3^.

In their recent paper^4^, Marek et al. evaluated the effects of sample size on univariate and multivariate BWAS in three large-scale neuroimaging datasets and came to the general conclusion that “BWAS reproducibility requires samples with thousands of individuals”. We applaud their comprehensive analysis, and we agree that (1) large samples are needed when conducting univariate BWAS and (2) multivariate BWAS reveal substantially larger effects and are therefore more highly powered.

Marek et al.^4^ find that multivariate BWAS provide inflated in-sample associations that often cannot be replicated (that is, are underpowered) unless thousands of participants are included. This implies that effect-size estimates from the discovery sample are necessarily inflated. However, we distinguish between the effect-size estimation method (in-sample versus cross-validated) and the sample (discovery versus replication), and show that, with appropriate cross-validation, the in-sample inflation that Marek et al.^4^ report in the discovery sample can be entirely eliminated. With additional analyses, we demonstrate that multivariate BWAS effects in high-quality datasets can be replicable with substantially smaller sample sizes in some cases. Specifically, applying a standard multivariate prediction algorithm to functional connectivity in the Human Connectome Project yielded replicable effects with sample sizes of 75–500 for 5 of 6 phenotypes tested (Fig. 1).

**Fig. 1:**
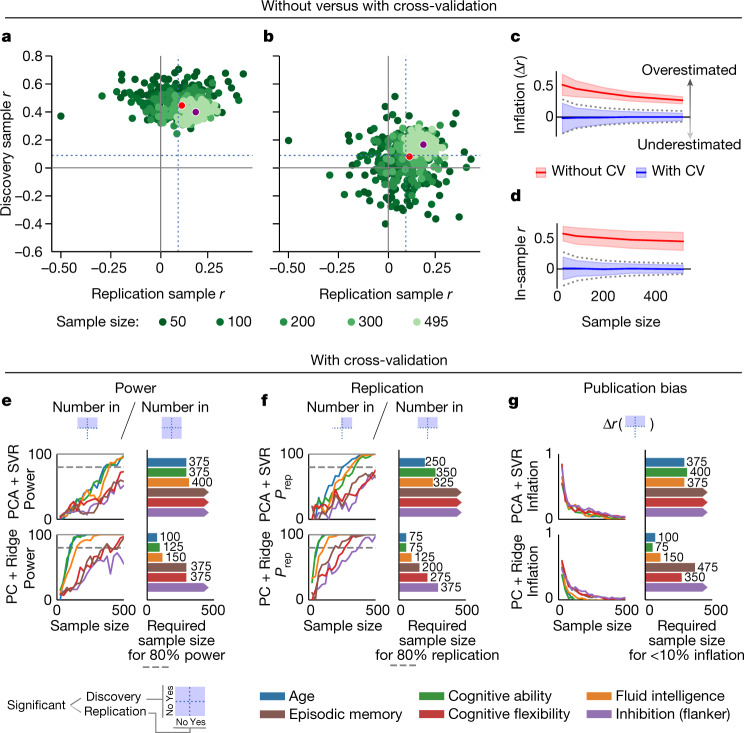
Examples of multivariate BWAS providing unbiased effect sizes and high replicability with low to moderate sample sizes. **a**, Discovery sample effects in multivariate BWAS are inflated only if estimates are obtained without cross-validation (CV). **b**, Cross-validation fully eliminates in-sample effect-size inflation and, as a consequence, provides higher replicability. Data are from the Human Connectome Project (HCP1200, PTN release, *n* = 1,003). Each point in **a** and **b** corresponds to one bootstrap subsample, as in figure 4b of Marek et al.^4^. The dotted lines denote the threshold for *P* = 0.05 with *n* = 495. Mean multivariate brain–behavioural phenotype associations across 100 bootstrap samples at *n* = 200 and for the full sample are denoted by red and purple dots. **c**, The inflation of in-sample effect size obtained without cross-validation (red) is reduced, but does not disappear, at higher sample sizes. Conversely, cross-validated estimates (blue) are slightly pessimistic with low sample sizes and become quickly unbiased as sample size is increased. **d**, Without cross-validation, in-sample effect-size estimates are non-zero (*r* ≈ 0.5, red), even when predicting permuted outcome data. Cross-validation eliminates systematic bias across all sample sizes (blue). The dashed lines in **c** and **d** denote 95% parametric confidence intervals, and the shaded areas denote bootstrap- and permutation-based confidence intervals. **e**,**f**, Cross-validated analysis reveals that sufficient in-sample power (**e**) and out-of-sample replication probability (*P*_rep_) (**f**) can be achieved for a variety of phenotypes at low or moderate sample sizes. 80% power and *P*_rep_ are achievable in <500 participants for 3 out of 6 phenotypes (coloured bars) using the prediction algorithm of Marek et al.^4^ (**e** and **f** (top), the sample size required for 80% power or *P*_rep_ is shown). The remaining three phenotypes require sample sizes of >500 (bars with arrows). Power and *P*_rep_ can be substantially improved with a ridge regression-based model recommended in some comparison studies^10,11^ (**e** and **f** (bottom), with 80% power and *P*_rep_ with sample sizes as low as *n* = 100 and *n* = 75, respectively, when predicting cognitive ability, and sample sizes between 75 and 375 for other investigated variables (fluid intelligence, episodic memory and cognitive flexibility), except inhibition assessed with the flanker task, which replicated with *n* = 375 but did not reach 80% power with *n* = 500. **g**, We estimated interactions between sample size and publication bias by computing effect size inflation (*r*_discovery_ − *r*_replication_) only for those bootstrap cases in which prediction performance was significant (*P* > 0.05) in the replication sample. Our analysis shows that the effect-size inflation due to publication bias is modest (<10%) with fewer than 500 participants for half of the phenotypes using the model from Marek et al.^4^ and all phenotypes but the flanker using the ridge model. The blue squares show conditional relationships assessed to derive metrics in **e**,**f** and **g** with reference to **b**. The top and bottom squares indicate positive and negative results in the discovery sample, respectively. The left and right squares indicate negative and positive results in the replication sample. The blue squares indicate how these conditions were applied to derive the metrics.

These analyses are limited to a selected number of phenotypes in a relatively high-quality dataset (measured in a young adult population with a single scanner) and should not be overgeneralized. However, they highlight that the key determinant of sample size requirements is the true effect size of the brain–phenotype relationship and that, with proper internal validation, appropriate effect-size estimates and sufficiently large effects for moderately sized studies are possible.

Marek et al.^4^ evaluate in-sample effect-size inflation in multivariate BWAS by training various multivariate models in a ‘discovery sample’ and comparing the in-sample effect sizes (prediction–outcome correlation, *r*) estimated from the training sample to the performance in an independent replication sample. On the basis of a bootstrap analysis, with variously sized pairs of samples drawn randomly from the Adolescent Brain Cognitive Development study, the authors report a severe effect-size inflation of Δ*r* = −0.29 (average difference between the in-sample effect sizes in the discovery sample and the out-of-sample effect sizes in the replication sample) and conclude that “[e]ven at the largest sample sizes (*n* ≈ 2,000), multivariate in-sample associations remained inflated on average”.

The issue with claims of inflation is that the in-sample effect size estimates of Marek et al.^4^ were based on training multivariate models on the entire discovery sample, without cross-validation or other internal validation (as confirmed by inspection of the code and discussion with the authors). Such in-sample correlations are not valid effect-size estimates, as they produce a well-known overfitting bias that increases with model complexity^5^. Standard practice in machine learning is to evaluate model accuracy (and other performance metrics) on data independent of those used for training. In line with current recommendations for multivariate brain–behaviour analyses^6,7^, this is typically performed using internal cross-validation (for example, *k*-fold) to estimate unbiased effect sizes in a discovery sample, and (less commonly) further validating significant cross-validated effects in held-out or subsequently acquired replication samples^2,5^.

Using cross-validation to estimate discovery-sample effects impacts the pool of studies selected for replication attempts, the degree of effect-size attenuation in replication samples, and the sample size needed for effective replication and mitigation of publication bias. To demonstrate this and provide quantitative estimates of sample size requirements in multivariate BWAS, we analysed functional connectivity data from the Human Connectome Project^8^ (one of the datasets in Marek et al.^4^) using cross-validation to estimate discovery-sample effect sizes. As shown in Fig. 1a–d, cross-validated discovery effect-size estimates are unbiased (that is, not inflated on average), irrespective of the sample size and the magnitude of the effect. As expected, even with cross-validation, smaller sample sizes resulted in lower power (Fig. 1e) and increased variability in effect-size estimates across samples (Fig. 1c). Such variability is undesirable because it reduces the probability of independent replication (Fig. 1f). Moreover, selection biases—most notably, publication bias—can capitalize on such variability to inflate effect sizes in the literature (Fig. 1g). Although these effects of using small sample sizes are undesirable, they do not invalidate the use of multivariate BWAS in small samples, and publication biases can be mitigated by practices that, like internal cross-validation, are quickly becoming standards in the field^2,5^. These include preregistration, registered reports, reporting confidence intervals and the use of hold-out samples tested only once on a single, optimized model to avoid overfitting.

Given these considerations, we wondered how many participants are generally required for multivariate BWAS. The answer to this question depends on the reliability of both phenotypic and brain measures, the size of the effects linking them, the algorithm and model-selection steps used and the use cases for the resulting brain measures. For example, multivariate models trained on as few as 20 participants^9^ can have high reliability (ICC = 0.84)^10^, broad external validity and large effect sizes (Hedges *g* = 2.3)^11^ in independent samples (for example, more than 600 participants from 20 independent studies in ref. ^11^) when predicting behavioural states within-person rather than traits. In this case, the benefit of large samples is primarily in accurately estimating local brain weights (model parameters)^12^ rather than increasing out-of-sample accuracy. Here we performed functional connectivity-based multivariate BWAS with cognitive ability (the phenotype shown in figure 4 of Marek et al.^4^) and five other cognition-related example phenotypes selected at random and demonstrate that, even when predicting trait-level phenotypes, as Marek et al.^4^ did, sample sizes of 75–500 are sufficient in five out of six of cases that we tested (or three out of six cases using the prediction algorithm of Marek et al.^4^) to achieve high statistical power and replicability (for example, 80%) and to mitigate effect size inflation due to publication bias.

The basis for these estimates is shown in Fig. 1e–g. Using cross-validated discovery sample effect-size estimates, the multivariate BWAS model of Marek et al.^4^—principal-component-based reduction of bivariate connectivity followed by support vector regression (PCA + SVR)—showed 80% in-sample power and 80% out-of-sample replication probability (*P*_rep_) at *n* < 500 for three out of six phenotypes that we examined (age, cognitive ability and fluid intelligence). However, this model has been shown to be disadvantageous in some comparison studies^12,13^. We therefore performed the same power and sample-size calculations for a multivariate BWAS using another approach—ridge regression on partial correlation matrices with a default shrinkage parameter of 1 (PC + ridge; Supplementary Methods). Although this approach is still probably suboptimal^12,13^ (we avoided testing other models to avoid overfitting), it substantially improved the power (Fig. 1e (bottom)), independent replication probability ([*P*_rep_]; Fig. 1f (bottom)) and resistance to inflation due to publication bias (Fig. 1g (bottom)). Eighty per cent power and *P*_rep_ were achieved at sample sizes from 75 to 150 for age (included as a reference variable), cognitive ability and fluid intelligence, and sample sizes < 400 for all phenotypes except for inhibition measured by the flanker task (a measure that is known to have low reliability^14^).

Our results highlight, that the key determinant of sample size requirements is the true effect size of the brain–phenotype relationship, which subsumes the amount, quality, homogeneity and reliability of both brain and phenotypic measures, and the degree to which a particular brain measure is relevant to a particular phenotype. Effect sizes will probably vary widely across studies; for example, cortical thickness can also reliably predict 4 out of the 6 investigated phenotypes with *n* < 500, although with smaller effect sizes on average (functional connectivity, mean *r* = 0.2; cortical thickness, mean *r* = 0.1; Supplementary Fig. 2). Although our results were derived from a relatively high-quality dataset and used an algorithm expected to yield larger effect sizes than that of Marek et al.^4^, they are in agreement with analytical calculations showing that BWAS that explain more than 1% of the phenotype’s variance can be replicable with sample sizes below 1,000 (Supplementary Methods). For example, a model that explains *r*^2^ = 0.01 (1% of variance) achieves 80% power in a prospective replication with *n* = 801, and *r*^2^ = 0.02 achieves 80% power with *n* = 399 (ref. ^15^).

These quantitative differences in required sample size could translate into large, qualitative differences in the types of neuroimaging studies considered viable in future efforts. There is a necessary trade-off between the innovativeness of a task, measure or method, and the extent to which it has been validated. Existing large-scale neuroimaging studies (*n* > 1,000) have selected well-validated tasks and imaging measures over new, exploratory ones, and few have attempted to characterize rare populations. Requiring sample sizes that are larger than necessary for the discovery of new effects could stifle innovation.

We agree with Marek et al.^4^ that small-sample studies are important for understanding the brain bases of tasks and mental states^9–11^, and for prototyping new tasks and measures. Furthermore, several current trends may further increase the viability of small-sample multivariate BWAS, including (1) new phenotypes, (2) feature-learning methods and algorithms with larger effect sizes^13^, (3) models that target within-person variation in symptoms and behaviour to improve between-person predictions^2^ and (4) hybrid strategies for improving prediction like meta-matching^16^. All of these have the potential to improve reliability and effect sizes, but whether they do remains to be seen.

Finally, as both Marek et al.^4^ and our analyses show, very small effects will suffer from limited power, replicability and predictive utility even with sample sizes in the thousands (Fig. 1). We argue that the field should focus on discovering phenotypes and brain measures with large effect sizes. Efficient discovery entails casting a wide net in smaller studies using rigorous, unbiased methods and scaling up promising findings to larger samples^2^. There are substantial challenges ahead, including establishing broad generalizability across contexts, equity across subpopulations, and models with high neuroscientific validity and interpretability^17,18^. Addressing these challenges will require innovative new methods and measures.

## Reporting summary

Further information on experimental design is available in the Nature Portfolio Reporting Summary linked to this Article.

## Online content

Any methods, additional references, Nature Portfolio reporting summaries, source data, extended data, supplementary information, acknowledgements, peer review information; details of author contributions and competing interests; and statements of data and code availability are available at 10.1038/s41586-023-05745-x.

## Supplementary information

Supplementary Information Supplementary Figs. 1 and 2, Supplementary Methods and Supplementary References.

Reporting Summary

## Data Availability

Analysis is based on preprocessed data provided by the Human Connectome Project, WU-Minn Consortium (principal investigators: D. Van Essen and K. Ugurbil; 1U54MH091657) funded by the 16 NIH institutes and centres that support the NIH Blueprint for Neuroscience Research; and by the McDonnell Center for Systems Neuroscience at Washington University. All data used in the present study are available for download from the Human Connectome Project (www.humanconnectome.org). Users must agree to data use terms for the HCP before being allowed access to the data and ConnectomeDB; details are provided online (https://www.humanconnectome.org/study/hcp-young-adult/data-use-terms).

## Code availability

All analysis code used in the current study is available at GitHub (release v.1.0; https://github.com/spisakt/BWAS_comment).
