# Supplementary material for: Multivariate BWAS can be replicable with moderate sample sizes
Source: Nature. 2023 Mar 8;615(7951):E4–7. doi: 10.1038/s41586-023-05745-x (PMC9995263; doi:10.1038/s41586-023-05745-x)
Supplement: Supplementary file 1 — Supplementary Figs. 1 and 2, Supplementary Methods and Supplementary References. [file 41586_2023_5745_MOESM1_ESM.pdf]

nature portfolio

https://doi.org/10.1038/s41586-023-05745-x

Supplementary information

Multivariate BWAS can be replicable with moderate sample sizes

In the format provided by the authors and unedited

Supplementary Information

Replicable multivariate BWAS with moderate sample sizes

Tamas Spisak[1,2], Ulrike Bingel[2], Tor Wager[3]

[1] Institute of Diagnostic and Interventional Radiology and Neuroradiology, University Medicine Essen, Germany
[2] Center for Translational Neuro- and Behavioral Sciences, Department of Neurology, University Medicine Essen, Germany
[3] Department of Psychological and Brain Sciences, Dartmouth College, Hanover, NH, United States

ARISING FROM: Marek et al., Reproducible brain-wide association studies require thousands of individuals. https://doi.org/10.1038/s41586-022-04492-9 (2022)

Supplementary Figures

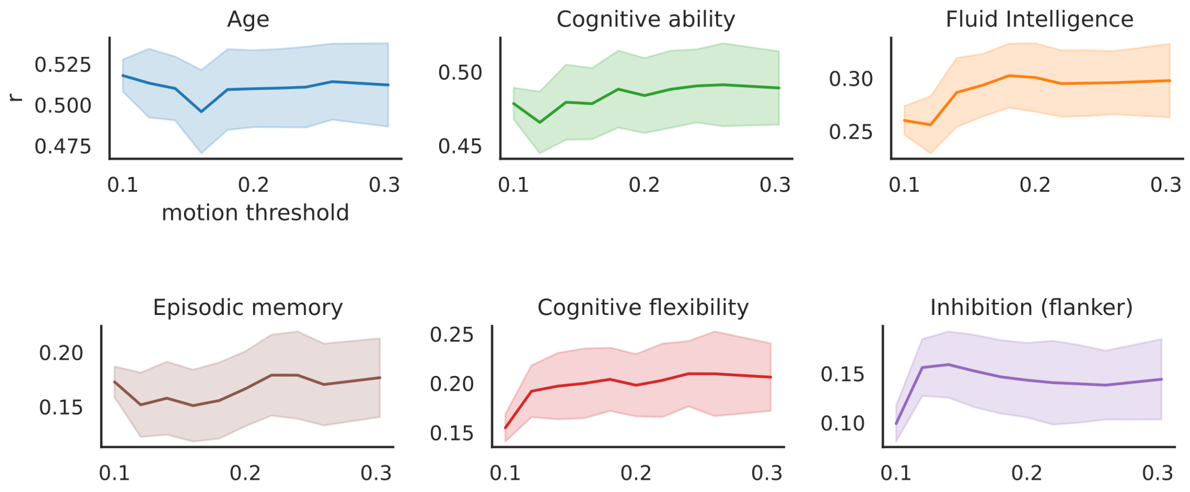

Supplementary Figure 1. *Dependence of out-of-sample predictive performance of the proposed multivariate approach as a function of in-scanner motion (mean root mean squared motion estimates) exclusion threshold, for all investigated variables. Sample size is equal for all motion thresholds (N=375 for both the discovery and replication samples; i.e. the number of participants passing the lowest threshold). Analysis was repeated 100 times on random samples (without replacement).*

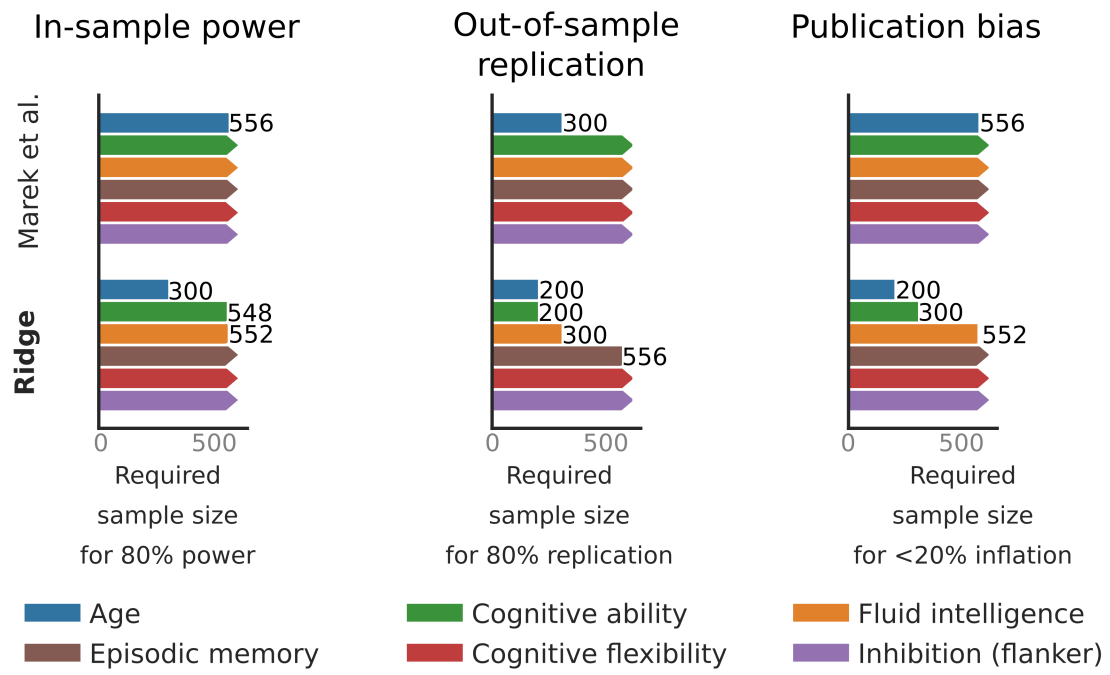

Supplementary Figure 2. *Cross-validated analysis based on cortical thickness measures revealed that sufficient in-sample power (left) and out-of-sample replication probability (P(rep)) (middle) can be achieved for a variety of phenotypes at low or moderate sample sizes. With Ridge regression, 80% power and P(rep) are achievable in <600 participants for age when using the prediction algorithm in Marek et al. (top panels in (e) and (f), sample size required for 80% power or P(rep) shown). Other phenotypes require sample sizes >600 (bars with arrows). Power and P(rep) can be substantially improved with a ridge regression-based model (bottom panels in (e) and (f)), with 80% power and P(rep) with sample sizes as low as n=548 and n=200, respectively, when predicting cognitive ability, and sample sizes between 200 and 556 for other investigated variables, except cognitive flexibility and inhibition assessed with the flanker task. We estimated interactions between sample size and publication bias (right) by computing effect size inflation ($r_{discovery}$ - $r_{replication}$) only for those bootstrap cases where prediction performance was significant (p>0.05) in the replication sample. Our results show that the effect size inflation due to publication bias is modest (<20%) with <500 participants for half the phenotypes using the Ridge model.*

Supplementary Methods

Functional connectivity Data

The Human Connectome Project dataset contains imaging and behavioral data of approximately 1200 healthy subjects[1]. Preprocessed resting state fMRI connectivity data (partial correlation matrices) as published with the HCP1200 release (N=999 participants with functional connectivity data) were used to build models that predict age, cognitive ability, episodic memory fluid intelligence, cognitive flexibility, and inhibition (flanker) scores.

Functional connectivity features were either (i) obtained via full Pearson's correlation coefficient or (ii) via partial correlation, across 100 group-independent component analysis based regions[2].

Cortical Thickness Data

Cortical thickness was analyzed with the 'Freesurfer'[3], in 63 regions, as defined in the 68 regions of the Desikan-Killiany Atlas[4].

Data Analysis

No participants were excluded due to high in-scanner motion. This decision was based on a bootstrap analysis of the dependence of out-of-sample predictive performance on the threshold for excluding participants. (Supplementary Figure 1).

Discovery and replication samples with various sample sizes were randomly sampled form all available participants 100 times for each sample size (ranging from 25 to ~500). On the discovery sample two machine learning models were evaluated via cross validation. The first model consisted of a principal component analysis retaining 50% of the total feature variance and a support vector regression and was trained on cortical thickness values and the full Pearson correlation features (as in Marek et al.). The second model was a Ridge regression (as implemented in the Python package 'Scikit-learn', with the default shrinkage parameter value of 1), trained on cortical thickness values (Supplementary Figure 2) and functional connectivity features.

Model performance in the discovery sample was evaluated by averaging the correlation coefficient between the predicted and observed values in the test set, across all folds.

The models were then fit on the whole discovery sample and used to predict the replication sample.

Analytical sample size calculations

Killeen's replication probability (p_{rep}) estimates the probability that a replication of an effect in a new sample will yield an effect of the same sign (no sign error). It depends on the effect size and degrees of freedom in the initial sample and the degrees of freedom in the replication sample, and can be calculated from the observed t-value in the initial sample, or the P-value with an additional assumption of known variance. The probability of obtaining a significant result in a replication sample at a given statistical threshold (e.g., $p < 0.05$) is termed p_{srep}, and is a straightforward extension. According to Eq. 6 of Ref.[5], Killeen's probability of significant replication[6] (p_{srep}) can be extended to unknown variance:

$$p_{srep}(\alpha) = \Pr\left(t_{rep} > T_\alpha \mid t_{obs}\right) = \Pr\left[K'_{(v,v)}(t_{obs}/\sqrt{2}) > T_\alpha/\sqrt{2}\right]$$

where K' is the K-prime distribution with its parameter v set to the degrees of freedom, t_{obs} and t_{rep} are the observed student-t values in the discovery and replication samples, respectively and T_α is the t-value threshold that must be surpassed for a successful replication

at an allowable false positive rate (significance-level) alpha. K-prime is a generalization of the noncentral t distribution, and in our case can be calculated from the initial sample t-value, degrees of freedom, and desired alpha level.

If statistical inference is based on Pearson's correlation (r; as in Marek et al.), the t-value for t_{obs} can be calculated as:

$$t_{obs} = r * \sqrt{\frac{n-2}{1-r^2}}$$

Based on the above equations, even if the correlation observed in the discovery sample is as low as r=0.1 (1% explained variance), at a sample size of n=1000, the probability of significant replication in a replication sample of the same size is:

$$p_{srep} = 0.86$$

From the same equation, sample sizes to achieve 80% replication probability with r2=0.02 (2% variance explained) and r2=0.01 (1% variance explained) are n=801 and 399, respectively.

Note that this approach is more conservative in sample size requirements than standard power analyses that assume a known, fixed population effect size. For example, with a known, true population effect size of $r^2 = 0.01$ (1% of variance), a replication study achieves 80% power with n = 616, and for $r^2 = 0.02$, 80% power is achieved with n = 307.

Analysis code is available at https://github.com/spisakt/BWAS_comment.

Supplementary References

1. Van Essen, D. C. et al. The wu-minn human connectome project: an overview. Neuroimage 80, 62–79 (2013).
2. Glasser, M. F. et al. The minimal preprocessing pipelines for the human connectome project. Neuroimage 80, 105–124 (2013).
3. Fischl B. FreeSurfer. Neuroimage. 2012 Aug 15;62(2):774-81.
4. Desikan RS, Ségonne F, Fischl B, Quinn BT, Dickerson BC, Blacker D, Buckner RL, Dale AM, Maguire RP, Hyman BT, Albert MS. An automated labeling system for subdividing the human cerebral cortex on MRI scans into gyral based regions of interest. Neuroimage. 2006 Jul 1;31(3):968-80.
5. Lecoutre B, Lecoutre MP, Poitevineau J. Killeen's probability of replication and predictive probabilities: How to compute, use, and interpret them. Psychological Methods. 2010 Jun;15(2):158.
6. Killeen, P. R. Predict, Control, and Replicate to Understand: How Statistics Can Foster the Fundamental Goals of Science. *Perspect. Behav. Sci.* **42**, 109–132 (2019).
