# Supplementary material for: Multivariate BWAS can be replicable with moderate sample sizes
Source: Nature. 2023 Mar 8;615(7951):E4–7. doi: 10.1038/s41586-023-05745-x (PMC9995263; doi:10.1038/s41586-023-05745-x)
Supplement: Supplementary file 2 — Reporting Summary [file 41586_2023_5745_MOESM2_ESM.pdf]

nature portfolio

Corresponding author(s): Tamas Spisak

Last updated by author(s): Dec 20, 2022

Reporting Summary

Nature Portfolio wishes to improve the reproducibility of the work that we publish. This form provides structure for consistency and transparency in reporting. For further information on Nature Portfolio policies, see our Editorial Policies and the Editorial Policy Checklist.

Statistics

For all statistical analyses, confirm that the following items are present in the figure legend, table legend, main text, or Methods section.

n/a	Confirmed	
☐	☒	The exact sample size (*n*) for each experimental group/condition, given as a discrete number and unit of measurement
☐	☒	A statement on whether measurements were taken from distinct samples or whether the same sample was measured repeatedly
☐	☒	The statistical test(s) used AND whether they are one- or two-sided<br>*Only common tests should be described solely by name; describe more complex techniques in the Methods section.*
☐	☒	A description of all covariates tested
☐	☒	A description of any assumptions or corrections, such as tests of normality and adjustment for multiple comparisons
☐	☒	A full description of the statistical parameters including central tendency (e.g. means) or other basic estimates (e.g. regression coefficient) AND variation (e.g. standard deviation) or associated estimates of uncertainty (e.g. confidence intervals)
☐	☒	For null hypothesis testing, the test statistic (e.g. F, t, r) with confidence intervals, effect sizes, degrees of freedom and P value noted<br>*Give P values as exact values whenever suitable.*
☒	☐	For Bayesian analysis, information on the choice of priors and Markov chain Monte Carlo settings
☒	☐	For hierarchical and complex designs, identification of the appropriate level for tests and full reporting of outcomes
☐	☒	Estimates of effect sizes (e.g. Cohen's *d*, Pearson's *r*), indicating how they were calculated

Our web collection on statistics for biologists contains articles on many of the points above.

Software and code

Policy information about availability of computer code

Data collection	Analysis is based on preprocessed data provided by the Human Connectome Project, WU-Minn Consortium (principal investigators: D. Van Essen and K. Ugurbil; 1U54MH091657) funded by the 16 NIH institutes and centers that support the NIH Blueprint for Neuroscience Research; and by the McDonnell Center for Systems Neuroscience at Washington University. All data used in the present study are available for download from the Human Connectome Project (www.humanconnectome.org). Users must agree to data use terms for the HCP before being allowed access to the data and ConnectomeDB; details are provided at https://www.humanconnectome.org/study/hcp-young-adult/data-use-terms. Preprocessed data was created software as described in:<br>Glasser MF, Sotiropoulos SN, Wilson JA, Coalson TS, Fischl B, Andersson JL, Xu J, Jbabdi S, Webster M, Polimeni JR, Van Essen DC. The minimal preprocessing pipelines for the Human Connectome Project. Neuroimage. 2013 Oct 15;80:105-24.
Data analysis	https://github.com/spisakt/BWAS_comment v1.0<br>Dependencies:<br>python 3.10.8 numpy 1.23.5 pandas 1.5.2 scikit-learn 1.2.0 joblib 1.2.0 mlxtend 0.21.0 seaborn 0.12.1 matplotlib 3.6.2

For manuscripts utilizing custom algorithms or software that are central to the research but not yet described in published literature, software must be made available to editors and reviewers. We strongly encourage code deposition in a community repository (e.g. GitHub). See the Nature Portfolio guidelines for submitting code & software for further information.

Data

Policy information about availability of data

All manuscripts must include a data availability statement. This statement should provide the following information, where applicable:

- Accession codes, unique identifiers, or web links for publicly available datasets
- A description of any restrictions on data availability
- For clinical datasets or third party data, please ensure that the statement adheres to our policy

Analysis is based on preprocessed data provided by the Human Connectome Project, WU-Minn Consortium (principal investigators: D. Van Essen and K. Ugurbil; 1U54MH091657) funded by the 16 NIH institutes and centers that support the NIH Blueprint for Neuroscience Research; and by the McDonnell Center for Systems Neuroscience at Washington University. All data used in the present study are available for download from the Human Connectome Project (www.humanconnectome.org). Users must agree to data use terms for the HCP before being allowed access to the data and ConnectomeDB; details are provided at https://www.humanconnectome.org/study/hcp-young-adult/data-use-terms.
All derivative data, (including raw material for figures) is freely available at https://github.com/spisakt/BWAS_comment

Human research participants

Policy information about studies involving human research participants and Sex and Gender in Research.

Reporting on sex and gender	The Human Connectome Project involved 656 females and 550 males. Findings apply to both males and females. Sex and gender differences are out of the scope of the Matters Arising and were not considered in the analysis. For more information, refer to van Essen et al., 2013.
Population characteristics	Refer to van Essen et al., 2013.
Recruitment	As described in van Essen et al., 2013.
Ethics oversight	As described in van Essen et al., 2013.

Note that full information on the approval of the study protocol must also be provided in the manuscript.

Field-specific reporting

Please select the one below that is the best fit for your research. If you are not sure, read the appropriate sections before making your selection.

☐ Life sciences ☒ Behavioural & social sciences ☐ Ecological, evolutionary & environmental sciences

For a reference copy of the document with all sections, see nature.com/documents/nr-reporting-summary-flat.pdf

Behavioural & social sciences study design

All studies must disclose on these points even when the disclosure is negative.

Study description	Quantitative analyses of the replicability of functional connectivity-based brain-wise association studies.
Research sample	We re-analyzed one of the studies involved in the original publication by Marek, Tervo-Clemmens et al., including open access-data from the Human Connectome Project (van Essen et al., 2013), based on a non-representative sample of young adults (656 females, 550 males, mean+-sd age: 28.9+-3.57).
Sampling strategy	We re-analyzed one of the studies involved in the original publication by Marek, Tervo-Clemmens et al., including open access-data from the Human Connectome Project. For more information, please refer to van Essen et al., 2013 ands Marek, Tervo-Clemmens et al, 2022.
Data collection	See van Essen et al., 2013 ands Marek, Tervo-Clemmens et al, 2022 for details.
Timing	See van Essen et al., 2013 ands Marek, Tervo-Clemmens et al, 2022 for details.
Data exclusions	We excluded participants with no MRI images available, and for each analysis, participants with missing data about the target phenotype.
Non-participation	203 out of the 1206 HCP participants didn't have MRI data. Exclusion die to missing phenotype data varied across analyses (less than 20 in all cases)
Randomization	N/A (observational study)

Reporting for specific materials, systems and methods

We require information from authors about some types of materials, experimental systems and methods used in many studies. Here, indicate whether each material, system or method listed is relevant to your study. If you are not sure if a list item applies to your research, read the appropriate section before selecting a response.

Materials & experimental systems

n/a	Involved in the study
☒	☐ Antibodies
☒	☐ Eukaryotic cell lines
☒	☐ Palaeontology and archaeology
☒	☐ Animals and other organisms
☒	☐ Clinical data
☒	☐ Dual use research of concern

Methods

n/a	Involved in the study
☒	☐ ChIP-seq
☒	☐ Flow cytometry
☐	☒ MRI-based neuroimaging

Magnetic resonance imaging

Experimental design

Design type: resting state fMRI, anatomical MRI

Design specifications: 4, 15min, eyes open (see van Essen et al., 2013)

Behavioral performance measures: As described in van Essen et al., 2013.

Acquisition

Imaging type(s): functional, anatomical

Field strength: 3

Sequence & imaging parameters: As described in van Essen et al., 2013.

Area of acquisition: brain

Diffusion MRI ☐ Used ☒ Not used

Preprocessing

Preprocessing software: As described in:
Glasser MF, Sotiropoulos SN, Wilson JA, Coalson TS, Fischl B, Andersson JL, Xu J, Jbabdi S, Webster M, Polimeni JR, Van Essen DC. The minimal preprocessing pipelines for the Human Connectome Project. Neuroimage. 2013 Oct 15;80:105-24.

Normalization: As described in: van Essen et al., 2013.

Normalization template: As described in: van Essen et al., 2013.

Noise and artifact removal: As described in: van Essen et al., 2013.

Volume censoring: As described in: van Essen et al., 2013.

Statistical modeling & inference

Model type and settings: Multivariate predictive modelling, PCA+SVR and Ridge, cross-validation (see Supplementary Methods for details).

Effect(s) tested: Predictive performance (Pearson's r)

Specify type of analysis: ☒ Whole brain ☐ ROI-based ☐ Both

Statistic type for inference (See Eklund et al. 2016): N/A (predictive modelling)

Correction: N/A (predictive modelling)

Models & analysis

n/a	Involved in the study
[ ]	[x] Functional and/or effective connectivity
[x]	[ ] Graph analysis
[ ]	[x] Multivariate modeling or predictive analysis

Functional and/or effective connectivity	partial correlation
Multivariate modeling and predictive analysis	Targets: age, cognitive ability, episodic memory, fluid intelligence, cognitive flexibility, inhibition. Model: PCA +SVR (reproduction of Marek et al.'s model), Ridge regression (with the default hyperparameter value 1). The PCA+SVR model involved dimensionality reduction.<br>Features partial correlation values across 100 ICA-based regions, training metric: mean squared error, evaluation metric, mean squared error and Pearson correlation.
